# A Novel Two Mode-Acting Inhibitor of ABCG2-Mediated Multidrug Transport and Resistance in Cancer Chemotherapy

**DOI:** 10.1371/journal.pone.0005676

**Published:** 2009-05-24

**Authors:** Hui Peng, Zizheng Dong, Jing Qi, Youyun Yang, Yang Liu, Zhaomin Li, Junkang Xu, Jian-Ting Zhang

**Affiliations:** 1 Department of Pharmacology and Toxicology, University School of Medicine, Indianapolis, Indiana, United States of America; 2 IU Simon Cancer Center, Indiana University School of Medicine, Indianapolis, Indiana, United States of America; Auburn University, United States of America

## Abstract

**Background:**

Multidrug resistance (MDR) is a major problem in successful treatment of cancers. Human ABCG2, a member of the ATP-binding cassette transporter superfamily, plays a key role in MDR and an important role in protecting cancer stem cells. Knockout of ABCG2 had no apparent adverse effect on the mice. Thus, ABCG2 is an ideal target for development of chemo-sensitizing agents for better treatment of drug resistant cancers and helping eradicate cancer stem cells.

**Methods/Preliminary Findings:**

Using rational screening of representatives from a chemical compound library, we found a novel inhibitor of ABCG2, PZ-39 (N-(4-chlorophenyl)-2-[(6-{[4,6-di(4-morpholinyl)-1,3,5-triazin-2-yl]amino}-1,3-benzothiazol-2-yl)sulfanyl]acetamide), that has two modes of actions by inhibiting ABCG2 activity and by accelerating its lysosome-dependent degradation. PZ-39 has no effect on ABCB1 and ABCC1-mediated drug efflux, resistance, and their expression, indicating that it may be specific to ABCG2. Analyses of its analogue compounds showed that the pharmacophore of PZ-39 is benzothiazole linked to a triazine ring backbone.

**Conclusion/Significance:**

Unlike any previously known ABCG2 transporter inhibitors, PZ-39 has a novel two-mode action by inhibiting ABCG2 activity, an acute effect, and by accelerating lysosome-dependent degradation, a chronic effect. PZ-39 is potentially a valuable probe for structure-function studies of ABCG2 and a lead compound for developing therapeutics targeting ABCG2-mediated MDR in combinational cancer chemotherapy.

## Introduction

Multidrug resistance (MDR) is a major problem in successful treatment of cancers. Over-expression of some members of the ABC (ATP-binding cassette) transporter superfamily has been suggested to cause MDR. P-glycoprotein (MDR1/ABCB1), multidrug resistance protein 1 (MRP1/ABCC1), and breast cancer resistance protein (BCRP/ABCG2) are three major ABC transporters that are major players in the clinical development of MDR [1]. One of these members, ABCG2 which is thought to exist and work as homo-oligomers of 8–12 subunits [2], [3], [4], has also been implicated to play roles in protecting cancer stem cells, resulting in drug resistance and failure of cancer chemotherapy [5]. Anticancer drug substrates of ABCG2 include but are not limited to the commonly used anticancer drugs such as Adriamycin, mitoxantrone, and topotecan. Indeed, recent clinical studies have shown that over-expression of ABCG2 in both adult and childhood leukemia correlates very well with poor prognosis (for a review see [6]). Knockout of ABCG2 had no apparent adverse effect on the development, biochemistry, and life of the mice [7]. All these previous observations make ABCG2 an ideal target for development of chemo-sensitizing agents for better treatment of drug resistant cancers and suggest that inhibiting ABCG2 unlikely will cause any side effect if the inhibitor is specific to ABCG2.

Compared with the well known drug resistance-causing ABC transporters such as ABCB1 and ABCC1, ABCG2 was discovered relatively recently and, thus, few specific inhibitors of ABCG2 have been reported. One of the known specific ABCG2 inhibitors is the potent mycotoxin Fumitremorgin C (FTC) secreted from *Aspergillus fumigatus* [8], [9]. However, the neurotoxicity of FTC limits its therapeutic potential. Analogues of FTC, such as Ko132 and Ko143, have been developed with low toxicity [10], but not yet known if effective in clinical trials. In addition, other inhibitors of ABCG2 have been reported [11], [12]. However, these agents, such as GF120918, appear to lack specificity due to their effect on ABCB1 and/or ABCC1 [13], [14]. Clearly, more specific ABCG2 inhibitors are needed for future development of potential chemo-sensitizers to better treat drug resistant cancers.

In this paper, we report discovery of a novel specific ABCG2 inhibitor, PZ-39 (N-(4-chlorophenyl)-2-[(6-{[4,6-di(4-morpholinyl)-1,3,5-triazin-2-yl]amino}-1,3-benzothiazol-2-yl) sulfanyl]acetamide), which is much more effective in reversing ABCG2-mediated drug resistance and less cytotoxic to cultured cells compared with FTC. PZ-39 appears to have two modes of actions by causing ABCG2 degradation (chronic) in addition to inhibiting its activity (acute). The similar effect of three PZ-39 related compounds revealed structural basis for the design of more potent specific ABCG2 inhibitors in the future.

## Results

### Effect of compound PZ-39 on mitoxantrone accumulation

**Figure 1:**
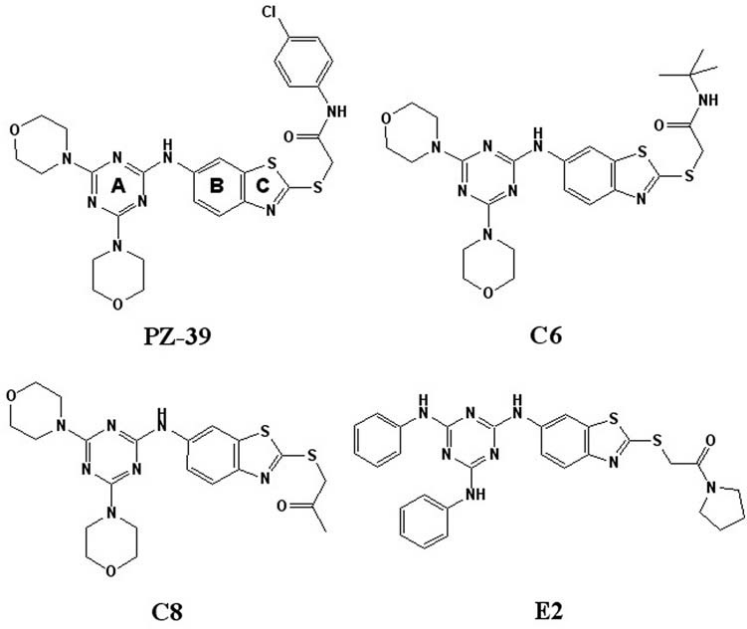
Schematic 2-dimensional chemical structures of PZ-39 and related compounds. PZ-39, N-(4-chlorophenyl)-2-[(6-{[4,6-di(4-morpholinyl)-1,3,5-triazin-2-yl]amino}-1,3-benzothiazol-2-yl)sulfanyl]acetamide, and its analogues C6, C8 and E2 all contain a benzothiazole linked to a triazine ring backbone. The three intact rings are labeled as A, B and C.

**Figure 2:**
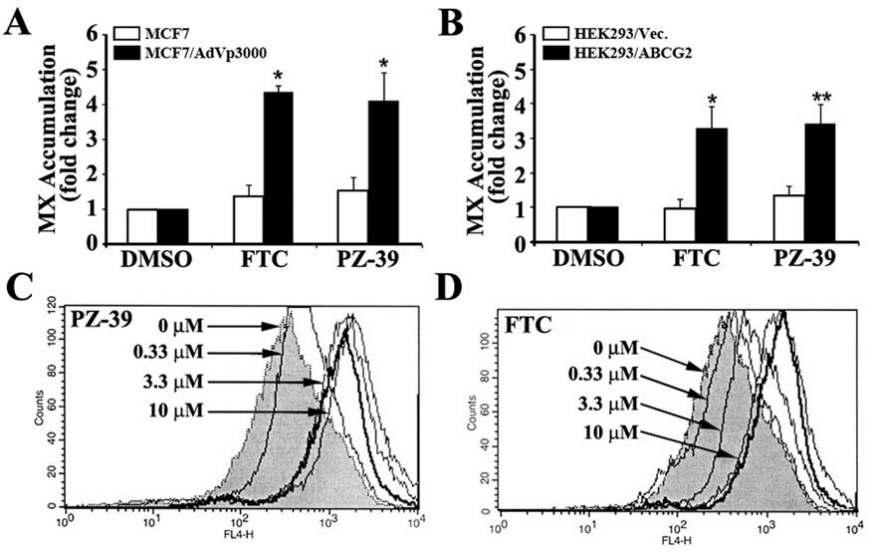
Effect of PZ-39 on intracellular mitoxantrone accumulation. A and B, mitoxantrone accumulation in MCF7 or its drug-resistant subline MCF7/AdVp3000 (A) and HEK293 cells transfected with vector or ABCG2 (B) following a 30 minute incubation in the absence or presence of PZ-39 (3.3 µM) or FTC (10 µM). The data are means±SD from three independent experiments (*P<0.05; **P<0.01 compared with DMSO vehicle). C and D, dose response of PZ-39 and FTC in restoring mitoxantrone accumulation in ABCG2-transfected HEK293 cells. The thick line shows the level of mitoxantrone accumulation in vector-transfected HEK293 cells, serving as a control.

Using a rational screening of representatives of different classes of a small molecule compound library from Specs (www.specs.net) for potential inhibitors of ABCG2-mediated drug efflux, we identified a compound, N-(4-chlorophenyl)-2-[(6-{[4,6-di(4-morpholinyl)-1,3,5-triazin-2-yl]amino}-1,3-benzothiazol-2-yl)sulfanyl]acetamide) with benzothiazole linked to triazine ring backbone, (named PZ-39 thereafter, see Fig. 1) that drastically reversed mitoxantrone accumulation in MCF7/AdVp3000 cells that over-express ABCG2. As shown in Fig. 2A, PZ-39 enhanced mitoxantrone accumulation in MCF7/AdVp3000 but not the parental sensitive MCF7 cells that do not produce ABCG2, suggesting that ABCG2-mediated drug efflux has been inhibited. Because MCF7/AdVp3000 cells also over-express other ABC transporters such as ABCC3 in addition to ABCG2 [15], PZ-39 may inhibit ABC transporters other than ABCG2, leading to increased mitoxantrone accumulation. To directly test if PZ-39 inhibits ABCG2, we performed similar studies using ABCG2-transfected stable HEK293 (HEK293/ABCG2) cells [3]. As shown in Fig. 2B, pre-incubation of cells with PZ-39 enhanced intracellular mitoxantrone accumulation in HEK293/ABCG2 but not in the vector-transfected control (HEK293/Vec) cells. Thus, PZ-39 likely inhibits ABCG2-mediated mitoxantrone efflux. Figs. 2A and 2B also show that PZ-39 at 3.3 µM achieved equivalent level of effect to the known specific ABCG2 inhibitor FTC at 10 µM, suggesting that PZ-39 may be ∼3 times more potent than FTC.

To further investigate the potency of PZ-39 for ABCG2, the dose response effect of PZ-39 on mitoxantrone accumulation in HEK293/ABCG2 cells were determined using flow cytometry. As shown in Fig. 2C, the intracellular mitoxantrone level was increased by PZ-39 in a dose-dependent manner. At 3.3 µM, PZ-39 completely restored intracellular mitoxantrone level in HEK293/ABCG2 cells. FTC, on the other hand, achieved similar level of effect only at 10 µM (Fig. 2D), supporting the argument that PZ-39 is more potent than FTC (see above).

### Sensitization of drug resistance by PZ-39

**Figure 3:**
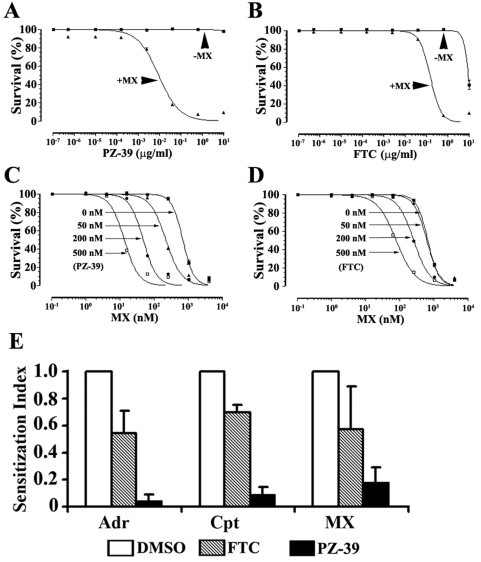
Effect of PZ-39 on sensitizing drug resistance. A and B, potency index of PZ-39 compared with FTC in reversing mitoxantrone resistance. HEK293/ABCG2 cells were treated without or with 0.1 µM (IC_10_) mitoxantrone in the absence or presence of different concentrations of PZ-39 (A) or FTC (B) followed by SRB assay. The data are a representative of four independent experiments. C and D, sensitization index of PZ-39 (C) compared with FTC (D) in HEK293/ABCG2 cells. HEK293/ABCG2 cells were treated with various concentrations of mitoxantrone in the absence or presence of different concentrations of PZ-39 followed by SRB assay. The data are a representative of four independent experiments. E, multidrug sensitization index of PZ-39 in drug-selected MCF7/AdVp3000 cells. MCF7/AdVp3000 cells were treated with various concentrations of adriamycin (Adr), camptothecin (Cpt), or mitoxantrone (MX) in the presence of DMSO (vehicle), 200 nM PZ-39, or FTC followed by SRB assay. Sensitization index was calculated using IC_50_ of mitoxantrone in the absence or presence of PZ-39 or FTC. The data shown are mean±SD of three independent experiments.

To investigate the potential use of PZ-39 as a chemo-sensitizer of ABCG2-mediated drug resistance, the effect of PZ-39 on drug response of HEK293/ABCG2 cells was determined in the absence or presence of 0.1 µM mitoxantrone which alone produced ∼10% cell killing. As shown in Fig. 3A and 3B, PZ-39 is not cytotoxic to HEK293/ABCG2 cells and its IC_50_ is not measurable within the concentration range used whereas the IC_50_ of FTC is ∼24 µM. The IC_50_ of PZ-39 and FTC required to sensitize mitoxantrone resistance is ∼15 nM and ∼387 nM, respectively. The potency index of PZ-39 is estimated to be >1600 whereas that of FTC is only ∼62 (Table 1).

**Table 1:** Potency index of PZ-39 in sensitizing drug resistance of HEK293/ABCG2 cells.

Inhibitors	IC_50_ (µM)		
	Inhibitor alone	Inhibitor+MX^a^	Potency Index^b^
PZ-39	>24^c^	0.015±0.005	>1600
FTC	24±1.04	0.387±0.054	62

a MX = mitoxantrone at 0.1 µM which produces ∼10% inhibition of growth (IC_10_).

b Potency Index = ratio of inhibitor IC_50_ in the absence and presence of anticancer drug mitoxantrone at low concentration (<IC_10_).

c PZ-39 has no measurable cytotoxicity within the concentration range used and its IC_50_ value was estimated to be bigger than that of FTC which is ∼24 µM.

To further investigate the inhibitory activity of PZ-39 on ABCG2, the effects of PZ-39 on mitoxantrone cytotoxicity in HEK293/ABCG2 cells were evaluated in the presence of three different concentrations of PZ-39 (50, 200, and 500 nM) or the vehicle control (0.1% DMSO). As shown in Fig. 3C, PZ-39 at 50 nM significantly reduced the IC_50_ of mitoxantrone with a sensitization index of 0.4 (Table 2). At 500 nM, the sensitization index of PZ-39 is 0.04 whereas that of FTC at the same concentration is 0.26 (Fig. 3D and Table 2). These results show that PZ-39 is a very potent novel ABCG2 inhibitor.

**Table 2:** Sensitization index of PZ-39 in HEK293/ABCG2 cells.

Inhibitors	Mitoxantrone IC_50_ (nM)		
	50 nM (SI^a^)	200 nM (SI)	500 nM (SI)
DMSO	551.1±20.8 (1)		
PZ-39	216.7±5.3 (0.39)	53.2±13.2 (0.10)	23.2±14.2 (0.04)
FTC	467.8±42.6 (0.85)	254.7±7.9 (0.46)	143.1±39.3 (0.26)

a SI = sensitization index, determined by dividing IC_50_ of mitoxantrone in the presence of inhibitors by IC_50_ of mitoxantrone in the presence of DMSO vehicle.

To investigate if PZ-39 can reverse ABCG2-mediated multidrug resistance in a drug resistant cancer cell line, we used the drug-selected MCF7/AdVp3000 cells and tested two additional anticancer drug substrates of ABCG2, Adriamycin and camptothecin. As shown in Fig. 3E, PZ-39 at 200 nM drastically reduced the resistance of MCF7/AdVp3000 to Adriamycin and Camptothecin similar to mitoxantrone, whereas the control FTC showed much less sensitization effect for all three drugs compared to PZ-39 at the same concentration.

### Two modes of action

**Figure 4:**
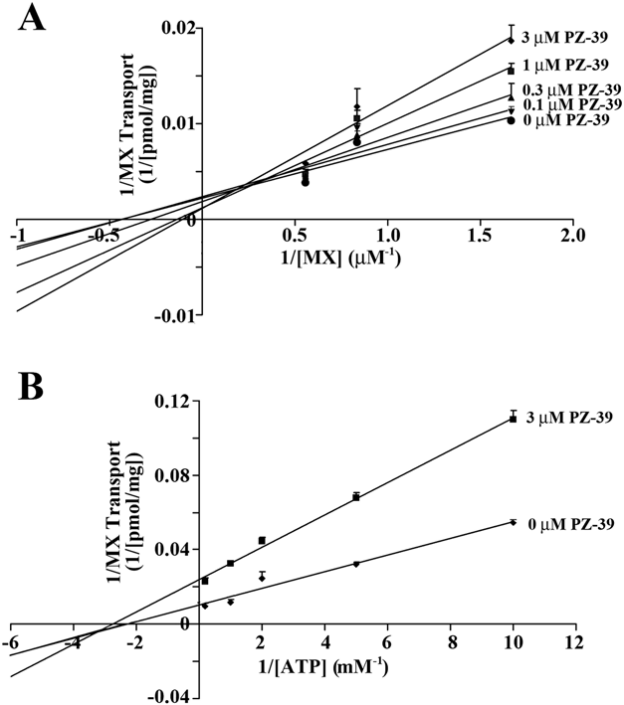
Kinetics of PZ-39 inhibition on ABCG2-mediated mitoxantrone uptake. Inside-out plasma membrane vesicles from HEK293/ABCG2 cells were incubated with 0.6, 1.2, and 1.8 µM [^3^H]mitoxantrone (A) or with 0.1, 0.2, 0.5, 1, and 5 mM ATP together with 0.6 µM [^3^H] mitoxantrone (B) in the absence or presence of different concentrations of PZ-39 at 37°C for 5 min followed by determination of mitoxantrone uptake. Data shown are mean±S.D. of three independent experiments.

To understand the mechanism of PZ-39 action in inhibiting ABCG2-mediated drug transport, we first investigated the kinetics of PZ-39 inhibition using isolated inside-out membrane vesicles [16]. We determined the change in mitoxantrone uptake in the presence of different concentrations of PZ-39. As shown in the Lineweaver-Burk plot (Fig. 4A), the Km and Vmax of mitoxantrone transport in the absence of PZ-39 were estimated to be 2.3 µM and 455 pmol/mg protein, respectively. It appears that both the Km and Vmax of mitoxantrone transport have been decreased in the presence of PZ-39 with an estimated Ki of ∼0.52 µM, suggesting that PZ-39 behaves as a mixed-type inhibitor of mitoxantrone transport [17]. Because it was also originally speculated that some inhibitors would compete with the ATP-binding site on ABCG2 transporter, we performed another experiment using ATP as a varying substrate. As shown in Fig. 4B, both the Km and Vmax of mitoxantrone transport were also altered by PZ-39. Thus, our study showed that the process of mitoxantrone transport and ATP binding may be inhibited by PZ-39 with a mixed type of mechanism. Likely, PZ-39 binds to a different site on ABCG2 from both mitoxantrone and ATP, not competitive inhibitor of either one of them.

We next tested if PZ-39 possibly inhibits ABCG2 oligomerization since ABCG2 has been suggested to function as a homodimer or higher forms of oligomers and oligomerization may be used as target for therapeutic drug development [2], [3]. For this purpose, co-immunoprecipitation of two differentially tagged ABCG2 was performed as previously described [2] following a 6-hr treatment with PZ-39 at 3.3 µM. However, no effect of PZ-39 on ABCG2 co-immunoprecipitation was found (supplemental Fig. S1), suggesting that PZ-39 does not affect ABCG2 oligomerization.

**Figure 5:**
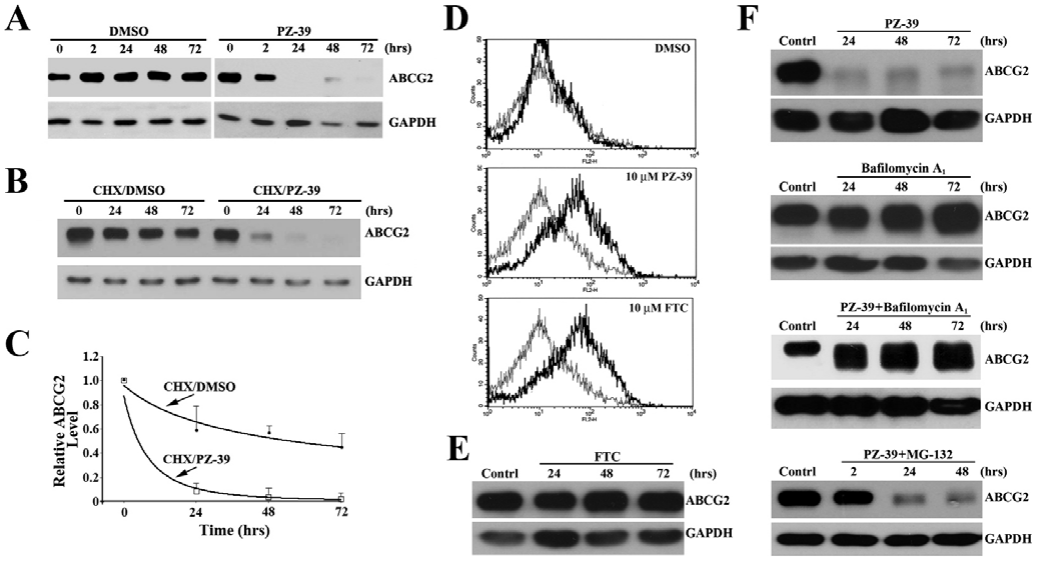
Effect of PZ-39 on ABCG2 expression, conformational change, and degradation. A, effect of PZ-39 on ABCG2 steady state level. HEK293/ABCG2 cells were treated with DMSO vehicle or 3.3 µM PZ-39 for various times and harvested for western blot analysis of ABCG2 expression. B, effect of PZ-39 on ABCG2 stability. HEK293/ABCG2 cells were first treated with cycloheximide (5 µg/ml) followed by addition of 3.3 µM PZ-39 or DMSO for various times and harvested for western blot analysis. C, half-life of ABCG2. ABCG2 levels on western blot as shown in B were determined using Scion Image and plotted against time of treatment. Data shown are mean±S.D of four experiments. D, effect of PZ-39 on 5D3 staining of ABCG2. HEK293/ABCG2 cells were treated without (thin line) or with (thick line) DMSO vehicle, 10 µM PZ-39 or FTC followed by staining with monoclonal antibody 5D3 and flow cytometry analysis. E, effect of FTC on ABCG2 expression. HEK293/ABCG2 cells were treated with 10 µM FTC for various times and harvested for western blot analysis of ABCG2 expression. F, effect of bafilomycin A_1_ and MG-132 on PZ-39-induced ABCG2 degradation. HEK293/ABCG2 cells were treated with 3 µM PZ-39 in the absence or presence of 10 nM Bafilomycin A_1_ or 2 µM MG-132 for various times and harvested for western blot analysis of ABCG2 expression. GAPDH was used as a loading control in all western blot analyses.

To further examine the mechanism of PZ-39 effect on ABCG2, we performed a western blot analysis of ABCG2 expression following PZ-39 treatment. As shown in Fig. 5A, the steady state level of ABCG2 protein drastically decreased at 1 day after PZ-39 treatment. But, it had only marginal decrease at 2 hours after PZ-39 treatment. As described above, PZ-39 was able to inhibit ABCG2-mediated mitoxantrone efflux of drug resistant cells with 1 hr of incubation. These findings suggest that PZ-39 may have two modes of action by inhibiting the activity (acute effect) and expression (chronic effect) of ABCG2 and consistent with our observation that PZ-39 is about 3 times better than FTC in drug accumulation assay (acute effect) but ∼7 times more potent in drug sensitization assay (chronic effect).

To determine if the chronic effect of PZ-39 on ABCG2 expression is at the mRNA level, we performed real-time RT-PCR analysis of MCF7/AdVp3000 and HEK293/ABCG2 cells treated with PZ-39 for various times up to 3 days. No significant change was found in ABCG2 mRNA level following PZ-39 treatment in either cell line (see supplemental Fig. S2). Thus, PZ-39 unlikely affects ABCG2 expression at its mRNA level. We next hypothesized that the mechanism of PZ-39-mediated inhibition would be a posttranscriptional process and examined the possibility that PZ-39 may accelerate the degradation of ABCG2. To test this possibility, HEK293/ABCG2 cells were pre-treated with cycloheximide, which acts by inhibiting elongation during protein synthesis, followed by treatment with PZ-39 for various times to determine ABCG2 degradation rate. As shown in Fig. 5B, the loss of ABCG2 in cells treated with a combination of PZ-39 and cycloheximide was much faster than that of the control treatment without PZ-39. The half-life of ABCG2 in the presence of PZ-39 is estimated to be ∼5 hrs whereas it is stable with an estimated half-life of ∼54 hrs in the control (Fig. 5C). Thus, PZ-39 likely accelerates the degradation of ABCG2 protein.

### Effect of PZ-39 on ABCG2 conformation and stability

The accelerated degradation of ABCG2 by PZ-39 may be due to that PZ-39 induces conformational change and target ABCG2 for degradation. To determine if PZ-39 potentially causes conformational changes of ABCG2, we used the monoclonal antibody 5D3 which has been reported previously to bind to ABCG2 on cell surface more readily in the presence of ABCG2 inhibitors presumably due to inhibitor-induced conformational changes [12], [18]. As shown in Fig. 5D, PZ-39 caused an increase in 5D3 staining, suggesting a possible conformational change of ABCG2 upon PZ-39 binding. FTC also increased 5D3 staining as expected. However, it did not affect the level of ABCG2 (Fig. 5E), suggesting that the conformational change induced by FTC and PZ-39 may be different. It has been reported recently that two distinct pathways exist for degradation of wild-type and mutant ABCG2 proteins [19]. While wild-type and correctly-folded protein is degraded in lysosomes, the mutant and misfolded protein is involved in ubiquitin-mediated protein degradation in proteasomes. To further determine the mechanism of PZ-39-induced ABCG2 degradation, we employed Bafilomycin A_1_, an inhibitor of protein degradation in lysosomes, and MG-132, a proteosome inhibitor. As shown in Fig. 5F, co-treatment of cells with Bafilomycin A_1_ and PZ-39 inhibited PZ-39-induced ABCG2 degradation whereas co-treatment with MG-132 and PZ-39 did not, indicating that PZ-39-induced ABCG2 degradation is likely lysosome-dependent. Taken together, we conclude that PZ-39 causes conformational change of ABCG2 and targets it for normal degradation in lysosomes.

### Effect of PZ-39 on ABCB1- or ABCC1-mediated drug transport

To determine the specificity of PZ-39, we tested the effect of PZ-39 on the other two important ABC transporters well known in MDR, ABCB1 and ABCC1. The effect of PZ-39 on ABCB1 and ABCC1-mediated decrease in intracellular Adriamycin accumulation was tested using MCF7 cells-transfected with ABCB1 (BC19) [20] and HEK293 cells-transfected with ABCC1 (HEK293/ABCC1) [16], [21]. We found no effect of PZ-39 on the activity of ABCB1 and ABCC1 in decreasing Adriamycin accumulation (see supplemental Fig. S3A). We also found no effect of PZ-39 on the steady state protein level of ABCB1 and ABCC1 after 3 days of treatment (Fig. S3B). Thus, likely PZ-39 is specific to ABCG2 among these three well known MDR-causing ABC transporters that are believed to play important roles in clinical drug resistance.

### Effect of PZ-39 analogues on ABCG2

**Figure 6:**
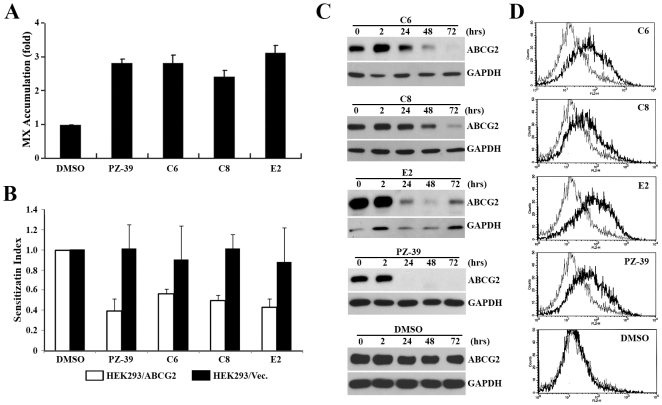
Effect of PZ-39 analogues on the function and expression of ABCG2. A, effects of PZ-39 and its analogue compounds (3 µM) on mitoxantrone accumulation in HEK293/ABCG2 cells. Data shown are mean±S.D. of triplicate experiments. B, sensitization index of PZ-39 and related compounds in HEK293/ABCG2 cells. HEK293/ABCG2 cells were treated with various concentrations of mitoxantrone in the absence or presence of 50 nM PZ-39, C6, C8, and E2 followed by SRB assay. Sensitization index was calculated using IC_50_ of mitoxantrone in the absence or presence of compound inhibitors. The data are mean±S.D. of four independent experiments. C, effect of selected PZ-39 and related compounds on ABCG2 steady state level. HEK293/ABCG2 cells were treated with DMSO vehicle or 3 µM PZ-39, C6, C8, or E2 for various times and harvested for western blot analysis of ABCG2 expression. D, effect of PZ-39 and related compounds on 5D3 staining of ABCG2. HEK293/ABCG2 cells were treated without (thin line) or with (thick line) DMSO vehicle, 10 µM PZ-39 or its related compounds C6, C8, and E2 followed by staining with monoclonal antibody 5D3 and flow cytometry analysis.

To better understand the pharmacophore of PZ-39, we apprehended 3 analogues of PZ-39 for drug accumulation and resistance assays using HEK293/ABCG2 cells in comparison with PZ-39. These analogues (C6, C8, and E2) all have the same intact rings A, B and C as PZ-39 but with different side groups (Fig. 1). All three analogues had similar activity as PZ-39 in enhancing mitoxantrone accumulation (Fig. 6A) and sensitizing mitoxantrone resistance in HEK293/ABCG2 (Fig. 6B). To determine the chronic effect of these analogues on ABCG2 expression, we performed a western blot analysis following treatment with C6, C8, and E2 for various times up to 3 days. As shown in Fig. 6C, all three analogues caused the decreased expression of ABCG2. Furthermore, all these analogues also caused conformational changes in ABCG2 similar to PZ-39 (Fig. 6D). Thus, likely the benzothiazole linked to a triazine ring backbone may be the core structure for binding to and inhibiting ABCG2 function and stability.

## Discussion

In this study, we investigated a novel potent specific inhibitor of human ABCG2, PZ-39, as a potential therapeutic agent to sensitize drug resistance in cancer chemotherapy. PZ-39 contains benzothiazole linked to a triazine ring backbone. Its mechanism of action appears to be in two modes; mixed type inhibition in drug transport function and accelerated lysosome-dependent degradation of ABCG2. PZ-39 is not cytotoxic itself with an IC_50_ of >24 µM while being very potent in sensitizing MDR of cancer cells over-expressing ABCG2.

Many previously reported ABCG2 inhibitors have a broad-spectrum of ABC transporter targets. ABCG2 inhibitor GF120918, for example, in fact, inhibits ABCB1 function more potently than ABCG2. Until now, very few compounds have been identified as specific inhibitors of ABCG2. One such example is the non-toxic FTC derivative, Ko143. It is more potent than other FTC analogues, and has no toxicity in mice at 10–50 mg/kg oral dose [10]. Recently, two of the flavone compounds, 6-prenylchrysin and tectochrysin, have been shown to be specific for ABCG2 and no interaction was detected with either ABCB1 or ABCC1 [22]. Using high throughput screening, Henrich et al. found several compounds that have similar or less inhibitory activity compared with FTC [11], [12]. Nevertheless, none of these reported specific ABCG2 inhibitors has been tested clinically.

Compared to some of the past known specific ABCG2 inhibitors, such as FTC, the novel compound PZ-39 has three distinctive advantages. First, PZ-39 is much more potent than FTC in inhibiting ABCG2 function. In the drug accumulation assay, PZ-39 clearly achieved the same level of inhibition at 3.3 µM compared with FTC at 10 µM. In cell survival assay, PZ-39 at 500 nM was able to sensitize ABCG2-mediated mitoxantrone resistance with an index of 0.04 whereas FTC at the same concentration has a sensitization index of 0.26, ∼7-fold difference. Second, PZ-39 has very low intrinsic cytotoxicity in vitro (>24 µM) but its potency index is much better than FTC (1600 versus 62). Thus, the window of therapeutic index of PZ-39 may be large. An ideal chemo-sensitizer is that it should not be toxic itself. Clearly, PZ-39 satisfies this requirement in in-vitro studies. However, future studies are needed to evaluate the toxicity of PZ-39 in animal models. Third, PZ-39 appears to have two modes of action. In addition to its ability to inhibit ABCG2 activity, PZ-39 also accelerates its lysosome-dependent degradation. This second mode of action is a distinctive nature and clearly increases the potency of PZ-39 on ABCG2 possibly by recycled use of PZ-39. This nature has not been reported for any previous known ABC transporter inhibitors.

The two modes of action make PZ-39 a very interesting, novel, and promising ABCG2 inhibitor for further exploitation. In the first mode of acute effect on ABCG2 function, PZ-39 appears to exert a mixed type of inhibition in drug uptake assay. PZ-39 may interact with ABCG2 directly, but do not appear to compete directly with mitoxantrone or ATP binding. Future studies are needed to identify the PZ-39 binding sites in ABCG2. In the second mode, PZ-39 appears to accelerate the ABCG2 degradation in lysosomes, a chronic effect. It is possible that binding of PZ-39 causes conformational change in ABCG2 which targets it for degradation in lysosomes. It is, however, noteworthy that binding of FTC also causes ABCG2 conformational change but it does no accelerate ABCG2 degradation. This finding suggests that the conformational change induced by PZ-39 and FTC may be different. Previously, it has been found that the cysteine mutant ABCG2 degradation is via proteosome whereas the normal degradation of wild type ABCG2 is via lysosome [19] with a half-life of ∼37 hrs [23]. It has also been found that the agonist-induced degradation of β_2_-adrenergic receptor is via lysosome possibly by enhanced endocytosis [24]. It is tempting to propose that the binding of PZ-39 to ABCG2 is able to accelerate the endocytosis and trafficking of cell surface ABCG2 into lysosomes for degradation. Extensive efforts to further evaluate this hypothesis are currently ongoing in our laboratory.

The analogues of PZ-39, C6, C8 and E2 with the same core structure all appear to work using the same mechanism as PZ-39. Clearly, further studies will be required to test more analogues of PZ-39 with more alterations on the core benzothiazole linked to a triazine ring backbone and to elucidate structure-activity relationship of PZ-39 in ABCG2 inhibition. Nevertheless, the observations from this study clearly indicate that PZ-39 may serve as a lead compound for further design and optimization of more specific ABCG2 inhibitors for better treatment of drug resistant human cancers in combinational therapy.

## Materials and Methods

### Materials

Monoclonal antibody BXP-21 against ABCG2, anti-Myc and anti-HA antibodies were from ID Labs, Cell Signaling, and Roche, respectively. Monoclonal antigody against Pgp, C219, was a kind gift from Dr. Victor Ling (The British Columbia Cancer Center, Vancouver, Canada). Monoclonal antibody against MRP, MRPr1, were purchased from Kamiya Biomedical Company. Biotin-conjugated 5D3 antibody and Phycoerythrin-Streptavidin conjugates were from eBiosciences. All electrophoresis reagents, protein concentration assay kit, precast polyacrylamide gradient gels and polyvinylidene difluoride membranes were purchased from Bio-Rad. FTC, adriamycin, mitoxantrone, camptothecin, DTT, Sulforhodamine B (SRB), and Triton X-100 were from Sigma. Protein-G PLUS-Agarose and SYBR Green PCR Master Mix were from Santa Cruz Biotechnology and Applied Biosystems, respectively. LipofectAMINE Plus and G418 were from Invitrogen. Cell culture medium IMEM, DMEM, and [^3^H]mitoxantrone were from BioSource International, Media Tech., and Moravek Biochemical, respectively. PZ-39 and three related compounds were purchased from SPECS. All other chemicals were of molecular biology grade from Sigma or Fisher Scientific.

### Cell culture, lysate, and membrane preparations

Human breast cancer cell line MCF7 (ATCC) and its derivative lines BC19 (a gift from Julie Horton at National Institute of Environmental Health Sciences) and MCF7/AdVp3000 (a gift from Susan Bates at National Cancer Institute), HEK293/ABCC1, HEK293/vector, HEK293/ABCG2 were cultured as previously described [2], [16], [20], [25]. Lysate preparation was performed as described previously [25]. Cell membranes were prepared in exactly the same way as previously described [16] and final membranes were resuspended in STBS (250 mM sucrose, 150 mM NaCl, 10 mM Tris/HCl, pH7.5).

### Western blot, immunoprecipitation, and flow cytometry

Western blot, immunoprecipitation, and flow cytometry analysis of drug accumulation were performed exactly as we previously described [2], [3]. To determine the mechanism of ABCG2 degradation, HEK293/ABCG2 cells were first treated with 10 nM Bafilomycin A_1_ or 2 µM MG132 for 24 hrs followed by additional treatment with 3 µM PZ-39 for various times. Cell lysates were then collected for western blot analysis of ABCG2. To determine the half-life of ABCG2, HEK293/ABCG2 cells were treated with 5 µg/ml cycloheximide, 3 µM PZ-39, or both for various times followed by collection of cell lysates for western blot analysis of ABCG2 expression. To determine the change in antibody 5D3 staining following treatment with inhibitors, HEK293/ABCG2 cells were incubated with 10 µM PZ-39, C6, C8, E2, or FTC at 37°C for 30 min before biotin-conjugated 5D3 antibody (1∶100 dilution) was added and incubated for 2 hrs. Then, the cells were washed 3 times and incubated with Phycoerythrin-Streptavidin for 30 min followed by washing for 3 times and analyzed by flow cytometry.

### Real time RT-PCR and Cytotoxicity assay

RNA extraction and real-time RT-PCR were performed as we described previously [25]. The sequences of ABCG2 primers are 5′-GGCTTTCTACCTGCACGAAAACCAGTTGAG-3′ (forward) and 5′-ATGGCGTTGAGACCAG-3′ (reverse). The sequences of GAPDH primers are 5′-AAGGACTCATGACCACAGTCCAT-3′ (forward) and 5′-CCATCACGCCACAGTTTCC-3′ (reverse). The relative ABCG2 RNA level (2^ΔCT^) treated with inhibitors was expressed as percentage of the control (in the presence of 0.1% DMSO) where ΔCT (threshold cycle) = (CT_ABCG2_-CT_GAPDH_).

Cytotoxicity was determined using SRB colorimetric assay as previously described [25]. The effect of compound inhibitors on drug resistance was determined by exposing cells to a range of concentrations of anticancer drugs such as mitoxantrone in the absence or presence of different concentrations of the inhibitor. The potency and sensitization index of the inhibitors were calculated as follows:
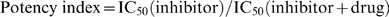


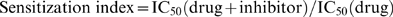



### Drug accumulation and transport kinetic analysis

Drug accumulation assay was performed as described previously [16], [26] with some modifications. Briefly, 10^6^ cells in culture were pre-incubated with various concentrations of PZ-39, FTC, or vehicle control (0.1% DMSO) for 1 hr at 37°C, followed by addition of 20 µM mitoxantrone and incubation for 30 min. The reaction was stopped by addition of ice-cold PBS and centrifugation, washed with ice-cold PBS, and subjected analysis flow cytometry.

Drug-uptake assay using membrane vesicles was performed as we previously described [16] using [^3^H]mitoxantrone as ABCG2 substrate. Kinetic analyses was performed using data generated in the presence of different concentrations of [^3^H]mitoxantrone, ATP, and PZ-39 to generate Lineweaver-Burk plot. Kinetic constants Km, Vmax, and Ki were calculated using procedures as previously described [27].

## Supporting Information

Figure S1 Effect of PZ-39 on ABCG2 oligomerization. HEK293 cells co-transfected with Myc- and HA-tagged ABCG2 were exposed to 3.3 µM PZ-39 for 6 hrs and cell lysates were subjected to immunoprecipitation with anti-Myc or anti-HA monoclonal antibody followed by western blot analysis probed using anti-HA and anti-Myc antibody.
(0.12 MB TIF)

Figure S2 Effect of PZ-39 on ABCG2 mRNA level. MCF7/AdVp3000 (A) and HEK293/ABCG2 (B) cells were treated with DMSO vehicle (open bar) or PZ-39 (filled bar) for various times and harvested for RNA preparation and real-time RT-PCR analysis. Data shown are mean±SD from three independent experiments.
(0.30 MB TIF)

Figure S3 Effect of PZ-39 on function and expression of ABCB1 and ABCC1. BC19 and HEK293/ABCC1 cells were treated with DMSO vehicle or 3.3 µM PZ-39 for 30 min followed by determination of intracellular accumulation of Adriamycin (A) or treated with DMSO vehicle or 3.3 µM PZ-39 for 3 days followed by western blot analysis of protein level (B). Thick lines represent control MCF7 cells transfected with vector for BC19 and HEK293 cells transfected with vector for HEK293/ABCC1. The gray areas and thick lines represent cells treated with DMSO and PZ-39, respectively. GAPDH was used as a loading control.
(0.57 MB TIF)
