# Supplementary figures and images for: A Novel Two Mode-Acting Inhibitor of ABCG2-Mediated Multidrug Transport and Resistance in Cancer Chemotherapy
Source: PLoS One. 2009 May 24;4(5):e5676. doi: 10.1371/journal.pone.0005676 (PMC2682573; doi:10.1371/journal.pone.0005676)

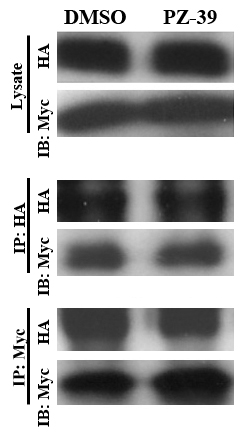

Supplement: Figure S1 — Effect of PZ-39 on ABCG2 oligomerization. HEK293 cells co-transfected with Myc- and HA-tagged ABCG2 were exposed to 3.3 µM PZ-39 for 6 hrs and cell lysates were subjected to immunoprecipitation with anti-Myc or anti-HA monoclonal antibody followed by western blot analysis probed using anti-HA and anti-Myc antibody. (0.12 MB TIF) [file pone.0005676.s001.tif]

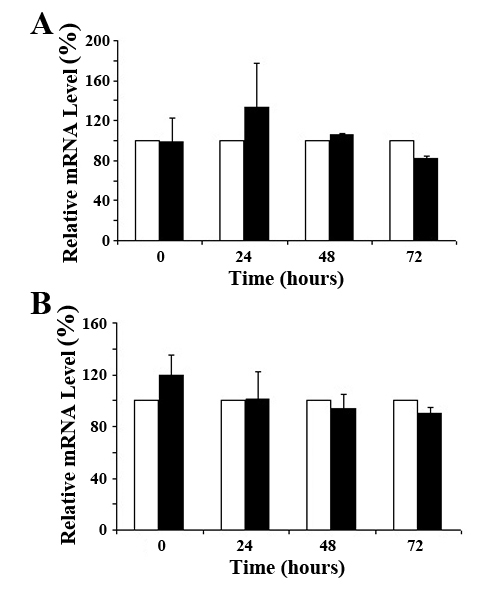

Supplement: Figure S2 — Effect of PZ-39 on ABCG2 mRNA level. MCF7/AdVp3000 (A) and HEK293/ABCG2 (B) cells were treated with DMSO vehicle (open bar) or PZ-39 (filled bar) for various times and harvested for RNA preparation and real-time RT-PCR analysis. Data shown are mean±SD from three independent experiments. (0.30 MB TIF) [file pone.0005676.s002.tif]

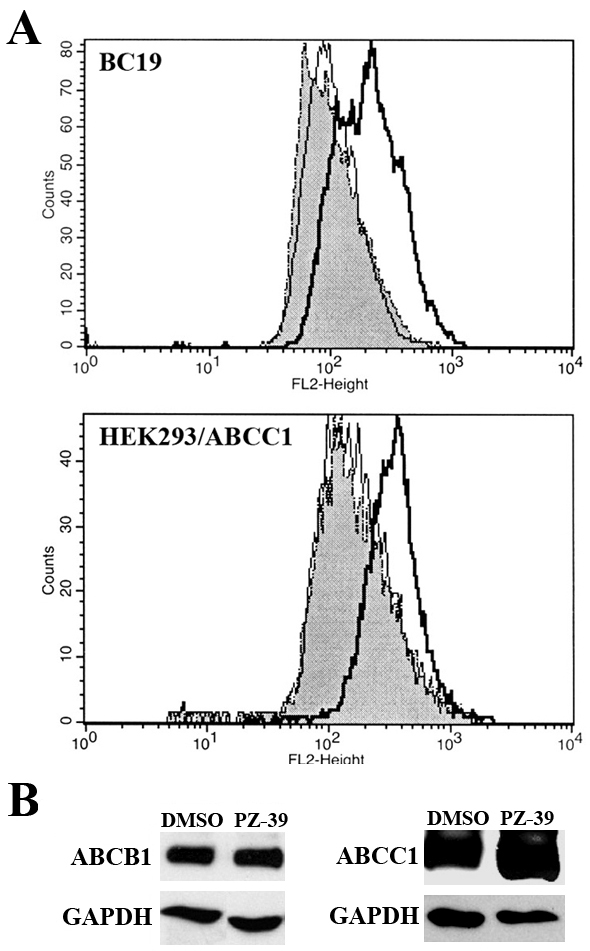

Supplement: Figure S3 — Effect of PZ-39 on function and expression of ABCB1 and ABCC1. BC19 and HEK293/ABCC1 cells were treated with DMSO vehicle or 3.3 µM PZ-39 for 30 min followed by determination of intracellular accumulation of Adriamycin (A) or treated with DMSO vehicle or 3.3 µM PZ-39 for 3 days followed by western blot analysis of protein level (B). Thick lines represent control MCF7 cells transfected with vector for BC19 and HEK293 cells transfected with vector for HEK293/ABCC1. The gray areas and thick lines represent cells treated with DMSO and PZ-39, respectively. GAPDH was used as a loading control. (0.57 MB TIF) [file pone.0005676.s003.tif]
